# A Haplotype of Two Novel Polymorphisms in δ-Sarcoglycan Gene Increases Risk of Dilated Cardiomyopathy in Mongoloid Population

**DOI:** 10.1371/journal.pone.0145602

**Published:** 2015-12-31

**Authors:** Jie Chen, Ye Jin, Hong Wang, Sisi Wei, Dan Chen, Li Ying, Qing Zhou, Gang Li, Joyce Li, Jimin Gao, Naoya Kato, Wei Hu, Yigang Li, Yuepeng Wang

**Affiliations:** 1 Molecular Cardiology Research Laboratory, Department of Cardiology, Affiliated Xinhua Hospital, Shanghai Jiaotong University School of Medicine, Shanghai, China; 2 Department of Ultrasound, Affiliated Xinhua Hospital, Shanghai Jiaotong University School of Medicine, Shanghai, China; 3 Ministry of Education and Shanghai Key Laboratory of Children’s Environmental Health, Affiliated Xinhua Hospital, Shanghai Jiaotong University School of Medicine, Shanghai, China; 4 Key Laboratory of Laboratory Medicine of the Chinese Ministry of Education, Wenzhou Medical College, Wenzhou, China; 5 Institute of Medical Science, University of Tokyo, Tokyo, Japan; 6 Department of Medicine, University of Massachusetts Medical School, Worcester, Massachusetts, United States of America; 7 Department of Cardiology, the Central Hospital of Minhang District, Fudan University School of Medicine, Shanghai, China; Rutgers University -New Jersey Medical School, UNITED STATES

## Abstract

The role of genetic abnormality of δ-sarcoglycan (δ-SG) gene in dilated (DCM) and hypertrophied (HCM) cardiomyopathy patients is still unfolding. In this study we first defined the promoter region and then searched for polymorphisms/mutations among the promoter, 5'-untranslated region, and the encoding exons in δ-SG gene in 104 Chinese patients with DCM, 145 with HCM, and 790 normal controls. Two novel polymorphisms were found, an 11 base-pair (bp) deletion (c._-100~-110_; -) in the promoter region and a missense polymorphism of A848G resulting in p.Q283R in the highly conserved C-terminus. The prevalence of homozygous genotype -/- of c._-100~-110_ was slightly higher in DCM (14.42%) and HCM patients (14.48%), as compared with normal controls (11.01%). The prevalence of genotype of 848A/G was significantly higher in DCM (6.73%; OR = 9.43; *p* = 0.0002), but not in HCM patients (1.38%; OR = 1.37; *p* = 0.62), as compared with controls (0.76%). Haplotype -_G consisting c._-100~-110_ and A848G was associated with increased risk of DCM (OR = 17.27; 95%CI = 3.19–93.56; *p* = 0.001) but not associated with HCM (OR = 1.90; 95%CI = 0.38–9.55; *p* = 0.44). Co-occurrence of the genotypes -/- of c._-100~-110_ and 848A/G was found in 5 patients with DCM (4.81%; OR = 39.85; *p* = 0.0001), none of HCM patients, and only 1 of the controls (0.13%). Both polymorphisms were also found in the Japanese population, but not in the Africans and Caucasians. C._-100~-110_ resulted in a decrease of δ-SG promoter activity to 64±3% of the control level (*p*<0.01). Both co-immunoprecipitation and *in vitro* protein pull-down assays demonstrated that δ-SG-283R interacts normally to β- and γ-SG, but significantly decreased localization of β/δ/γ-SG on the plasma membrane. In conclusion, haplotype -_G composed of c._-100~-110_ and A848G confers higher susceptibility to DCM in the Mongoloid population.

## Introduction

Cardiomyopathy (CM), one of the common causes of heart failure, arrhythmias, and mortality, is mainly divided into hypertrophic (HCM) and dilated cardiomyopathy (DCM) [1]. Genetic abnormalities account for about 70% of HCM and 30% of DCM [1–3]. Both types of CM are genetically heterogeneous. Inherited HCM mainly involves mutational genes encoding sarcomeric proteins [1–6], which are thought to alter the force generation by the sarcomere. Nearly 85% pathogenic mutations are from four genes encoding δ-myosin heavy chain, myosin-binding protein C, troponin T, and tropomyosin [5]. So far over 26 genes involving more than 400 loci have been reported and could account for about 90% of the inherited HCM [2, 4, 5].

The etiology genes of inherited DCM are still far from clarified. The thirty-three causative genes reported could explain only 30~35% of the total disease [1, 3, 7]. Titlin and Lamin A/C gene mutations have been shown to be the most common genetic abnormality [7–9]. About half of the genes encode sarcomeric proteins including α-cardiac actin, myosin heavy chain, troponin, tropomyosin, metavinculin, α-actinin and therefore overlap with those in HCM [1, 3, 7, 10]. DCM specific genes are those encoding cytoskeletal proteins, including dystrophin, sarcoglycan, metavinculin, desmin, Cypher/ZASP, α-Bcrystalin, and LIM domain protein-3 [7, 10]. Autosomal recessive, mithochondrial, and X-linked DCM have been described, but familial DCM is mainly transmitted as an autosomal dominant disease. However, clinically non-isolated forms of DCM represent only 10% of all familial DCM and majority of DCM patients carry sporadic and autosomal dominant mutations [10]. Given the fact that morbid mutations identified represent only a small percentage of familial DCM, it is reasonable to speculate that a large number of morbid genes remain to be discovered.

Of the above DCM specific genes encoding cytoskeletal proteins, dystrophin, sarcoglycan, and desmin belong to the dystrophin-associated glycoprotein complex (DGC). DGC is mainly composed of a trans-sarcolemmal glycoprotein subcomplex of α-, β-, γ-, and δ-sarcoglycan (SG), α- and β-dystroglycan (DG), and dystrophin [11]. Dystrophin serves as a link between intracellular F-actin and DGC, and by this architecture the intracellular mechanical force generated by muscle contraction can be transmitted to adjacent sarcomeres and to the extracellular matrix [11]. All four SG members are expressed in both heart and skeletal muscles and the genetic abnormalities of each member could result in autosomal-recessive limb-girdle muscular dystrophies (LGMD; LGMD-2D, -2E, -2C, and -2F, respectively) with different degree of cardiac involvement [12–14]. The LGMD patients with mutations in α-, β-, γ-, but not δ-SG gene usually associate with cardiac involvement [15, 16].

The role of δ-SG gene mutation in the pathogenesis of CM was first discovered in a Syrian hamster model of inherited CM and very mild muscular dystrophy [17]. In 1997, Sakamoto *et al* reported that a 30 kb deletion located in 5' upstream of the exon-2 of the gene was the direct etiology for the model [17]. Later in humans, Tsubata *et al* screened 50 young DCM patients (age 3 days–18 years) and one DCM family and detected two mutations in the δ-SG gene [18]. The p.S151A mutation was detected in three family members of one family and a 3bp deletion at position 238 (c.K238) in two sporadic cases. The p.S151A and c.K238 mutations caused a relatively severe form of DCM characterized by sudden cardiac death and heart failure at young age, but with no signs of LGMD-2F. Both mutations were autosomal dominant and the p.S151A was later proven to cause DCM in transgenic mice [19] and *Drosophila* [20], but did not cause DCM when it co-occurred with A131P, a LGMD-2F-causing mutation reported in German population [21]. In 2003, Karkkainen, *et al* reported the third causative p.R71T that also autosomal dominantly resulted in mild form of DCM without affecting the skeletal muscle in Finland population [22]. However, Sylvius, *et al* reported that δ-SG gene was only marginally implicated in French patients with DCM [23] and so did Date *et al* report in Japanese patients with HCM [24]. Since 2007, the mutation-searching studies have shifted to the 5'-untranslated region (5’UTR) of the gene. A G-94C polymorphism in the 5'UTR has been reported to be associated with the HCM in Japanese population [25]. The -94C allele was recently identified to be strongly associated with genetic susceptibility for HCM in Amerindians in Mexico as well [26, 27].

The inference that δ-SG could be an isolated etiology for CM is based only on the scarcity of above evidence and has been controversial and inconclusive [21]. On the other hand, there is no data on δ-SG mutation/polymorphism in Chinese CM patients. It is still unclear whether there is any CM-causing abnormality in the promoter region, as those reported in Syrian hamster model. Till now, no defined δ-SG promoter has been reported.

In this study, we first determined the promoter region of δ-SG gene and subsequently screened for single nucleotide polymorphism (SNP)/mutations in its promoter, the 5’UTR, and the coding regions in Chinese patients with DCM and HCM. Here we report two novel polymorphisms, one with an 11bp deletion (c._-100~-110_) in the promoter region and the other with a heterozygous missense polymorphism of A848G resulting in p.Q283R in exon-8. The prevalence of 848A/G genotype was 8.86 times higher in DCM and 1.81 times higher in HCM patients. Five of six cases with co-occurrence of the homozygous c._-100~-110_ and p.Q283R are found in DCM patients. Both polymorphisms are found only in the Mongoloid population. δ-SG-283R mildly but significantly decreased localization of β/γ/δ-SG on the plasma membrane. Our results suggested that the co-occurrence of homozygous c._-100~-110_ and p.Q283R contribute to the pathogenesis of DCM in eastern Asia countries.

## Methods

Additional details are available in S1 File: Expanded Methods.

### Subjects and DNA Samples

The study was approved by the institutional review board of Xinhua Hospital Affiliated to Shanghai Jiaotong University. Written informed consents for participation were obtained from all patients and healthy control subjects.

The patient pool consisted of 104 patients with DCM and 145 with HCM. None of the patients had symptoms of LGMD. The control group included 790 unrelated healthy Chinese. The clinical and echocardiography data are summarized in Table 1. Genomic DNA was extracted from whole peripheral blood. 459 cases of normal Japanese DNA samples were also included. Some of the normal DNA samples were purchased from the Coriell Cell Repositories (Camden, NJ).

**Table 1:** Clinical Data in DCM and HCM Patient and Control Groups

Index	DCM	HCM	Normals
**Number**	104	145	790
**Gender (M/F)**	65/39	107/38	427/363
**Age**	37±15	49±17	45±11
**IVS (mm)**	9±3	15±3	10±2
**PW (mm)**	9±2	14±2	10±2
**LVEDD (mm)**	57±8	45±6	46±5
**LVESD (mm)**	42±5	29±3	30±4
**FS (%)**	26±3	36±7	35±6
**LAD (mm)**	36±7	33±6	34±4
**Systolic BP (mmHg)**	114±11	123±14	115±15
**Diastolic BP (mmHg)**	74±5	78±7	76±6

### Amplification of Genomic Fragments, Allele and Genotype Analysis

**Fig 1:**
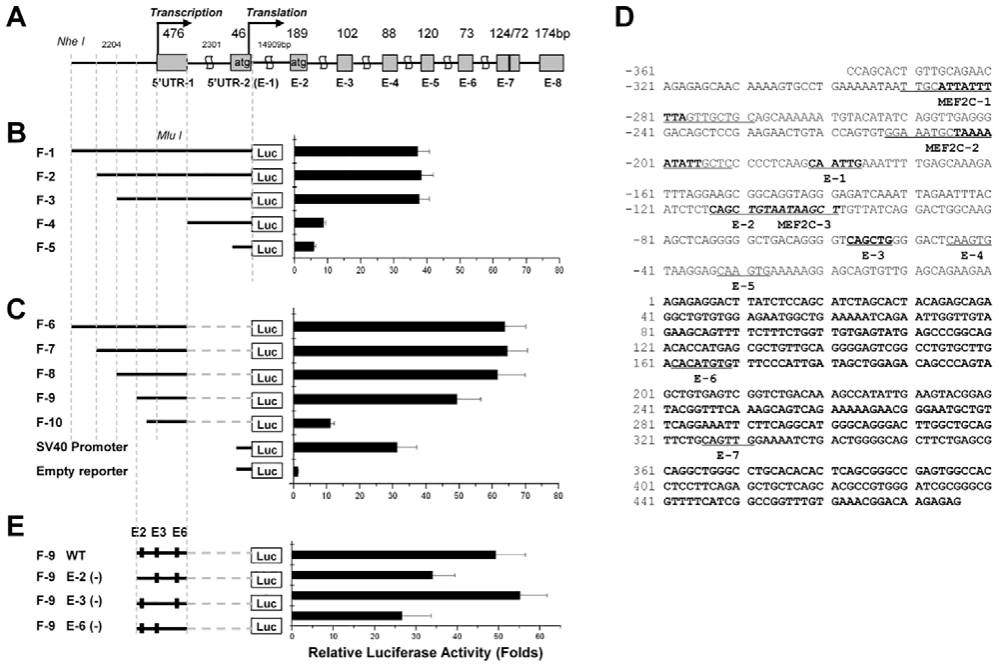
Determination of the human δ-SG gene promoter region. Fig 1*A* shows genomic structure of the human δ-SG gene. Fig 1B shows genomic fragment (F)-1~5 upstream of the translational start site and their corresponding luciferase activities. Fig 1C illustrates F-6~11 upstream of the end point of the 5’UTR-1, SV40 promoter, empty reporter, and their corresponding luciferase activities. Fig 1D is the 814bp sequence of the F-8 containing the promoter. The 5’-UTR starts from the +1 position and is shown in bold. The sequences of three putative MEF2C motifs and seven putative E-boxes (E) are shown in bold, underlined and indicated beneath of the each sequence. Fig 1E shows WT F-9 with putative E(2), E(3), and E(6) and those F-9 with serially-mutated E2/E3/E6 by site-directed mutagenesis (described in the text) and their corresponding luciferase activities.

The genomic structure of the human δ-SG gene is established in Fig 1A. Primers for amplification of the genomic fragments (F) and exons are listed in S2 and S3 Files. PCR products were subjected for sequencing or endonuclease digestion.

### Cell Culture

A rat skeletal muscle cell line (L6, JCRB 9801) and HEK-293T cells were cultured and passaged in Dulbecco’s modified Eagle’s medium (Gibco-Life) with 10% fetal bovine serum (Gibco-Life).

### Transient Expression and Firefly Luciferase Reporter Assay

Luciferase activities were measured in L6 cells with a dual-luciferase reporter assay system (Promega).

### Nuclear Protein Extraction (NPE)

Nuclear protein was extracted from the left ventricular myocardial tissue of golden hamster (S6 File).

### Electrophoretic Mobilized Shift Assay (EMSA)

Biotin-labeled and unlabeled DNA duplexes probes carrying myocyte-specific enhancer factor 2C (MEF2C) or E-box sequence (S4 File) were made. DNA-protein binding reactions were performed by incubating 10 ug NPE for 20 min with 2ug poly dI-dC (Amersham) and 200 fMol of each probes, resolved in 8% polyacrylamide gels, and transferred to Hybond-N^+^ membrane (Amersham, RPN303B).

### Cloning of DGC Components’ cDNA

The cDNAs representing either full length or extracellular domain of genes in DGC complex were amplified from a human heart cDNA library (Clontech, CA), and cloned into pcDNA3.1-myc/his(+)A or pGEX-4T-1 (Amersham Biosciences). The mutant of δ-SG-283R mutant cDNA was obtained by site-directed mutagenesis. Human Hand1, Hand2, and MEF2C’s cDNA were cloned into pQE-30.

### Antibodies

The monoclonal antibodies against α-; β-; γ-; and δ-SG; and β-DG were purchased from Novocastra (Newcastle upon Tyne, UK). The monoclonal antibodies against α-DG (Clone VIA4-1) were purchased from Millipore (Upstate, USA). Rabbit antibodies against HSP90 were purchased from ABclonal (Wuhan, China).

### Immnuoprecipitation

Each protein in DGC complex was over-expressed in HEK-273T cells, harvested, and immunoprecipitated with their cognant antibodies.

### *In vitro* Transcription-translation

*In vitro* transcription and translation was performed using the TNT kit (Promega, WI) as directed by the manufacturer.

### GST- and 6His-fusion Protein Expression and Purification

All GST- and 6His-fusion proteins were expressed in E. Coli and purified.

### Pull-down Assay

Fifty micro-liter reaction mixture containing 100ng of each translated α-, β-, and γ-SG and α- and β-DG proteins was mixed with 50μl of GST-δ-SG-CT-283Q or GST-δ-SG-CT-283R bound to beads.

### Cell Surface Biotinylation

HEK-293T cells were washed with PBS and treated with 1 mM membrane-impermeable NHS-LC-biotin in 50 mM HEPES (pH 7.4). After incubation at room temperature for 30 min, the reaction was quenched in 100 mM Tris-HCl (pH 7.4). The biotin-labeled cells were washed with PBS and total proteins were extracted with TLB buffer. The biotin-labeled proteins were isolated by incubating cell lysate with avidin-agarose. The flow-through fraction was collected and the avidin-agarose was then washed with PBS with 0.1% Tween-20 and the biotin-labeled proteins were eluted from the avidin-agarose with SDS sample buffer.

### *Western* Blotting

The proteins were separated by SDS-PAGE and electrophoretically transferred to Immobilon-P (Millipore, MA). Immunodetection was accomplished using the ECL *Western* blotting (WB) kit (Thermo-Pierce).

### Statistic Analysis

Genotype frequencies in cases and controls were compared using a χ^2^ test with Yates' correction. Odds ratio (OR) and 95% confidence interval (CI) were calculated by unconditional logistic regression analysis. Linkage disequilibrium (LD) between c._-100~-110_ and A848G was estimated in the control group using Lewontin’s standardized coefficient *D’* and LD coefficient *r* ^2^. The haplotypes were inferred from all genotype data, and haplotype analyses were conducted using SNPStats (http://bioinfo.iconcologia.net/snpstats/start.htm) [28]. All the statistical analyses were performed with the SPSS15.0 software, with two-sided tests and a significant level of *p* value <0.05.

## Results

### Localization of the Promoter Region of the Human δ-SG Gene and Identification of the Active E-boxes

In order to define the promoter region, we first conducted a luciferase assay with luciferase reporter constructs carrying different deletional fragments of 5’UTR of d-SG gene (F-1~10) in L6 cells. As shown in Fig 1B, the luciferase activities demonstrated a 37.2~38.2-fold increase for F-1, -2 and -3, but only a 5.8~8.8-fold increase for F-4 and -5. These results suggest that the promoter is likely located in the region between the F-4 and -1, an 814bp fragment including 476bp 5’UTR1 and its upstream 338bp. To further confirm the promoter region and rule out any possible effects of 2301bp interon-1 and 46bp 5’UTR-2 (exon-1) on the activity, we performed luciferase assay with F-6~10. As shown in Fig 1C, the reporter activities were increased 61.6~64.5-fold for F-6, -7, and -8, indicating that the interon-1 and 5’UTR-2 inhibit the reporter activity. We observed a 48-fold increase in the activity for deletional mutant F-9. However, further deletion (F-10) showed only an 11.1-fold increase, indicating that the high promoter activity came from the region between F-9 and -10. These results are consistent with those in Fig 1B. Taken together, we identified that F-8 (-338 to +476bp) represents the whole promoter region of human δ-SG gene.

Sequence analysis revealed that the above identified δ-SG promoter contains 3 putative MEF2C motifs (Fig 1D) with MEF2C-1, -2 highly conserved among species. However, as shown in Fig 1C, MEF2C-1 and -2 would only account for about 20% promoter activity by calculating the difference between F-8 and -9.

Further analysis identified 7 putative E-box (Fig 1D), with E-2, E-3, and E-6 are highly conserved. Based on the results shown in Fig 1, F-9 gave the highest luciferase activity and contains all 7 putative E-boxes without MEF2C-1 and -2 sites. We therefore chose F-9 construct for further mutagenesis analysis. To determine the key important binding motif, we performed serial mutations to delete those motifs. As shown in Fig 1E, we found that the E-2, E-3, and E-6 played a functional role in regulating δ-SG gene expression.

### C._-100~-110_ Located at Immediate Downstream of a Putative E-box

**Fig 2:**
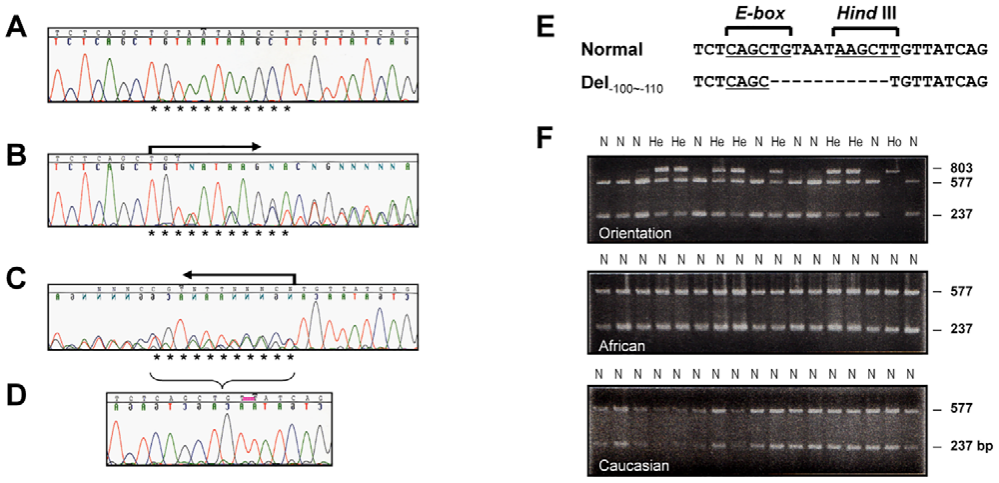
Genotypic analysis of the promoter-containing F-8. Fig 2*A* shows the normal genomic sequence of -90~-120 within the 5’-UTR. Fig 2*B* and 2*C* demonstrate the location (*) of the heterozygous deletion deduced from overlapped waves respectively in forward (B) and in reverse (C) directions (*arrows*). Fig 2*D* shows a homozygous c._-100~-110_. Fig 2*E* shows losing of a Hind *III* site due to the c._-100~-110_. Fig 2*F* illustrates differential cutting patterns of normal (*N*; double bands of 237 and 577bp), heterozygous (*He*; triple bands of 237, 577, and 803bp), and homozygous (*Ho*; single band of 803bp) c._-100~-110_ in F-8 in Orientation, African, and Caucasian populations. The cut samples were separated and visualized on 2% agarose ethidium bromide gel.

Next, we searched for SNP/mutations in the 814bp promoter region in CM patients by direct sequencing. In some samples, compared to the WT (Fig 2A), overlapped spikes always started from a certain area in both forward (Fig 2B) and reverse (Fig 2C) sequencing directions. These overlapping spikes disappeared when we sequenced a sample from a patient carrying an 11bp-deletion in both alleles. This indicated that the overlapped sequencing spikes was due to a heterozygous 11bp-deletion (c._-100~-110_; Chrom-5: 155,753,657–155,753,667; Fig 2D). C._-100~-110_ locates just downstream of the E-2 and this AT-rich sequence (TGTAATAAGCT) itself likely constitutes the third putative MEF2C binding motif (MEF2C-3). The c._-100~-110_ abolished unique Hind *III* site (Fig 2E), which was used as an easy identification in mass genotyping (Fig 2F).

As shown in Table 2, the genotype frequency of heterozygous c._-100~-110_ is 41.34% (43/104) in DCM, 46.21% (67/145) in HCM patients, with no significant difference compared to the Chinese normal controls (385/790; 48.73%). The frequency is lower (183/469; 39.02%) in Japanese population. However, the frequency of homozygous c._-100~-110_ show slightly higher in patients with DCM (15/104, 14.42%; OR = 1.36; *p* = 0.32) and HCM (21/145, 14.48%; OR = 1.37; *p* = 0.26), as compared with the controls (87/790, 11.01%). It is also lower (33/469, 7.04%) in the Japanese population. Both heterozygous and homozygous c._-100~-110_ were also found in a small population of Koreans and Thai. Surprisingly, neither was found in 110 Caucasians nor in 110 Africans.

**Table 2:** Alleles and Genotype Count with Their Frequencies of Del_-100~-110_ and A848G in Our Studied Population.

Ethnicity	Number	Allele Count		Genotype Count			Allele Count		Genotype Count			Compound Genotype Count			Compound Genotype Count		
		-	TGTAATAAGCT	-/-	-/TGTAATAAGCT	TGTAATAAGCT/TGTAATAAGCT	848G	848A	848G/G	848G/A	848A/A	-/-	-/TGTAATAAGCT	TGTAATAAGCT/TGTAATAAGCT	-/-	-/TGTAATAAGCT	TGTAATAAGCT/TGTAATAAGCT
												& 848G/A	& 848G/A	& 848G/A	& 848A/A	& 848A/A	& 848A/A
**DCM**	104	73 (35.10%)	135 (64.90%)	15 (14.42%)	43 (41.34%)	46 (44.23%)	7 (3.37%)	201 (96.63%)	0 (0%)	7 (6.73%)	97 (93.27%)	5 (4.81%)	1 (0.96%)	1 (0.96%)	10 (9.62%)	42 (40.38%)	45 (43.27%)
**HCM**	145	109 (37.59%)	181 (62.41%)	21 (14.48%)	67 (46.21%)	57 (39.31%)	2 (0.69%)	288 (99.31%)	0 (0%)	2 (1.38%)	143 (98.62%)	0 (0%)	1 (0.69%)	1 (0.69%)	21 (14.48%)	66 (45.52%)	56 (38.62%)
**Ctl Chinese**	790	559 (35.38%)	1021 (64.62%)	87 (11.01%)	385 (48.73%)	318 (40.25%)	6 (0.38%)	1574 (99.6%)	0 (0%)	6 (0.76%)	784 (99.24%)	1 (0.13%)	3 (0.38%)	2 (0.25%)	86 (10.89%)	382 (48.35%)	316 (40.00%)
** Japanese**	469	249 (26.54%)	689 (73.45%)	33 (7.04%)	183 (39.02%)	253 (53.94%)	4 (0.43%)	934 (99.6%)	0 (0%)	4 (0.85%)	465 (99.15%)	0 (0%)	2 (0.43%)	2 (0.43%)	33 (7.04%)	181 (38.59%)	251 (53.52%)
** Koreans**	12	7 (29.17%)	17 (70.83%)	1 (8.33%)	5 (41.67%)	6 (50.00%)	0 (0%)	24 (100%)	0 (0%)	0 (0%)	24 (100%)	0 (0%)	0 (0%)	0 (0%)	1 (8.33%)	5 (41.67%)	6 (50.00%)
** Thai**	13	9 (34.62%)	17 (65.38%)	1 (7.69%)	7 (53.85%)	6 (46.15%)	0 (0%)	26 (100%)	0 (0%)	0 (0%)	26 (100%)	0 (0%)	0 (0%)	0 (0%)	1 (7.69%)	7 (53.85%)	6 (46.15%)
** Caucasian**	110	0 (0%)	220 (100%)	0 (0%)	0 (0%)	110 (100%)	0 (0%)	220 (100%)	0 (0%)	0 (0%)	220 (100%)	0 (0%)	0 (0%)	0 (0%)	0 (0%)	0 (0%)	110 (100%)
** Africans**	110	0 (0%)	220 (100%)	0 (0%)	0 (0%)	110 (100%)	0 (0%)	220 (100%)	0 (0%)	0 (0%)	220 (100%)	0 (0%)	0 (0%)	0 (0%)	0 (0%)	0 (0%)	110 (100%)

Our results from both 790 normal Chinese and 469 normal Japanese are consistent with the data of the Asian population (286 including 97 Han Chinese in Beijing, 100 Southern Han Chinese, and 89 Japanese in Tokyo) in the “1000 Genomes Project” (Table 3). However, our data using both 110 Caucasians and 110 West Africans normal controls are slightly different from those in the “1000 Genomes Project”, which illustrates 2.0% heterozygous c._-100~-110_ in 246 Africans and 1.7% heterozygous c._-100~-110_ in 379 Europeans.

**Table 3:** Alleles and Genotypes with Their Frequencies of Del_-100~-110_ in 1092 People Worldwide in the “1000 Genomic Project”.

Ethnicity	Number	Allele Count		Genotype Count		
		-	TGTAATAAGCT	-/-	-/TGTAATAAGCT	TGTAATAAGCT/TGTAATAAGCT
**All**	1092	206 (9.4%)	1978 (90.6%)	31 (2.8%)	144 (13.2%)	917 (84.0%)
**East Asian (ASN)**	286	191 (33.4%)	381 (66.6%)	31 (10.8%)	129 (45.1%	126 (44.1%)
Han Chinese in Beijing	97	58 (29.9%)	136 (70.1%)	9 (9.3%	40 (41.2%)	48 (49.5%)
Southern Han Chinese	100	83 (41.5%)	117 (58.5%)	15 (15%)	53 (53.0%)	32 (32.0%)
Japanese in Tokyo	89	50 (28.1%	128 (71.9%)	7 (7.9%)	36 (40.4%)	46 (51.7%)
**African (AFR)**	246	5 (1.0%	487 (99.0%	0 (0%)	5 (2.0%)	241 (98%)
**Mixed American (AMR)**	181	3 (0.8%)	359 (99.2%)	0 (0%)	3 (1.7%)	178 (98.3%)
**European (EUR)**	379	7 (0.9%)	751 (99.1%)	0 (0%)	7 (1.8%)	372 (98.2%)

Within the promoter region of δ-SG, several other loci of SNPs (A108T; C148G; A217G; G426C) were found in both CM patients and normal Chinese controls. However, those SNPs frequency showed no significant difference among HCM, DCM, and controls (data not shown).

### A Novel Missense Polymorphism of A848G in the Highly Conserved Exon-8

**Fig 3:**
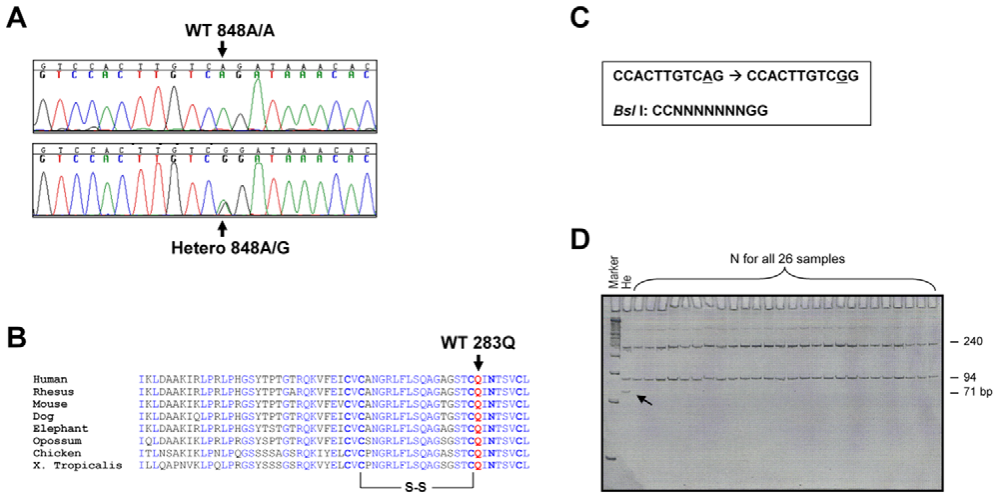
Genotypic analysis of the Exon-8 of the human δ-SG gene. Fig 3*A* shows genomic sequences of a wild type sample (*upper panel*) and a sample with 848A/G (*lower panel*). Fig 3*B* illustrates that 848A/G results in Q283A in the highly conserved C-terminus of the δ-SG gene among different species. Fig 3*C* shows that 848A/G creates a neonate cutting site for *Bsl* I. Fig 3*D* demonstrates differential cutting patterns by *Bsl* I of normal samples (*N*; double bands of 94 and 240bp) and a sample with 848A/G (*He*; quadruple bands of 23, 71, 94 and 240bp). The cut samples were separated on 12.5% polyacrylamide gel and the gels were stained by SYBR-green.

Next, we searched for SNP/mutations in all 8 exons in CM patients by direct sequencing. No previously-reported mutations or SNPs were found in exon-1~7 [18, 21–25]. In exon-8, a novel nucleotide substitution of A848G (CAG→CGG), resulting in conversion of p.Q283R (Fig 3A), was discovered in the universally conserved extreme C-terminus (Fig 3B). This polymorphism creates a new restriction enzyme site for *Bsl* I (Fig 3C), which we used for genotyping (Fig 3D).

This polymorphism is not on the list of the “1000 Genome Project”, indicating it is a novel mutation or a rare SNP. The genotype for both patients and controls were heterozygous. The genotype frequency of 848A/G was significant higher in DCM (7/104, 6.73%; OR = 9.43; *p* = 0.0002), as compared with HCM patients (2/145, 1.38%; OR = 1.37; *p* = 0.62) and controls (6/790; 0.76%; Table 4). This 848G allele was also found in Koreans, Japanese, and Thai population, but not in the Africans and Caucasians (Tables 2 and 3).

**Table 4:** Haplotype Analysis in DCM Patients *vs* Control Population.

Haplotype	Frequency		OR (95% CI)	P value
	Ctl	DCM		
TGTAATAAGCT_A	0.6442	0.6445	1	-
-_A	0.352	0.3225	0.87 (0.63–1.21)	0.41
-_G	0.0018	0.0284	17.27 (3.19–93.56)	0.001
TGTAATAAGCT_G	0.002	0.0052	2.57 (0.24–27.29)	0.43

OR, odds ratio; CI, confident interval

### Association between C._-100~-110_ and A848G

Obtained from LD analysis, *D’* is 0.1955 and *r* ^*2*^ is 0.00027 between c._-100~-110_ and A848G in the control group, indicating low LD between these two loci.

In DCM group, of 7 cases carrying 848A/G, 5 cases were concomitant with homozygous -/- of c._-100~-110_ and 1 case each with heterozygous -/TGTAATAAGCT and WT, respectively (Table 2). In HCM group, of 2 cases carrying 848A/G, 1 case each was in coincidence with heterozygous -/TGTAATAAGCT and WT, respectively. In the normal group, of 6 cases carrying 848A/G, 1 case was in coincidence with homozygous -/- of c._-100~-110_ and 3 and 2 cases were respectively with heterozygous -/TGTAATAAGCT and WT. Similarly, of 4 cases carrying 848A/G in healthy Japanese, none was concomitant with homozygous -/- of c._-100~-110_ and 2 cases each were with heterozygous -/TGTAATAAGCT and WT, respectively. Compared to the prevalence in normal Chinese group (0.13%), cases with co-occurred -/- of c._-100~-110_ and 848A/G have a significantly increased prevalence (4.81%) and a remarkably high specificity (OR = 39.85; *p* = 0.0001) in DCM patients.

The associations between the haplotypes composed of c._-100~-110_ and A848G and risk of DCM and HCM were summarized in Table 4 and S5 File, respectively. Haplotype -_G was significantly associated with increased risk of DCM (OR = 17.27; 95%CI = 3.19–93.56; *p* = 0.001) but not associated with HCM (OR = 1.90; 95%CI = 0.38–9.55; *p* = 0.44).

### Decrease in the Promoter Activity Caused by C._-100~-110_

We then studied the impact of c._-100~-110_ on the promoter activity. The luciferase activities were compared between WT, F-1, -6, -8, and -9 and matching mutants containing homozygous c._-100~-110_. As shown in Fig 3A, the c._-100~-110_ resulted in a significant decrease in the promoter activity to 64±3% of the control levels (*p*<0.01).

**Fig 4:**
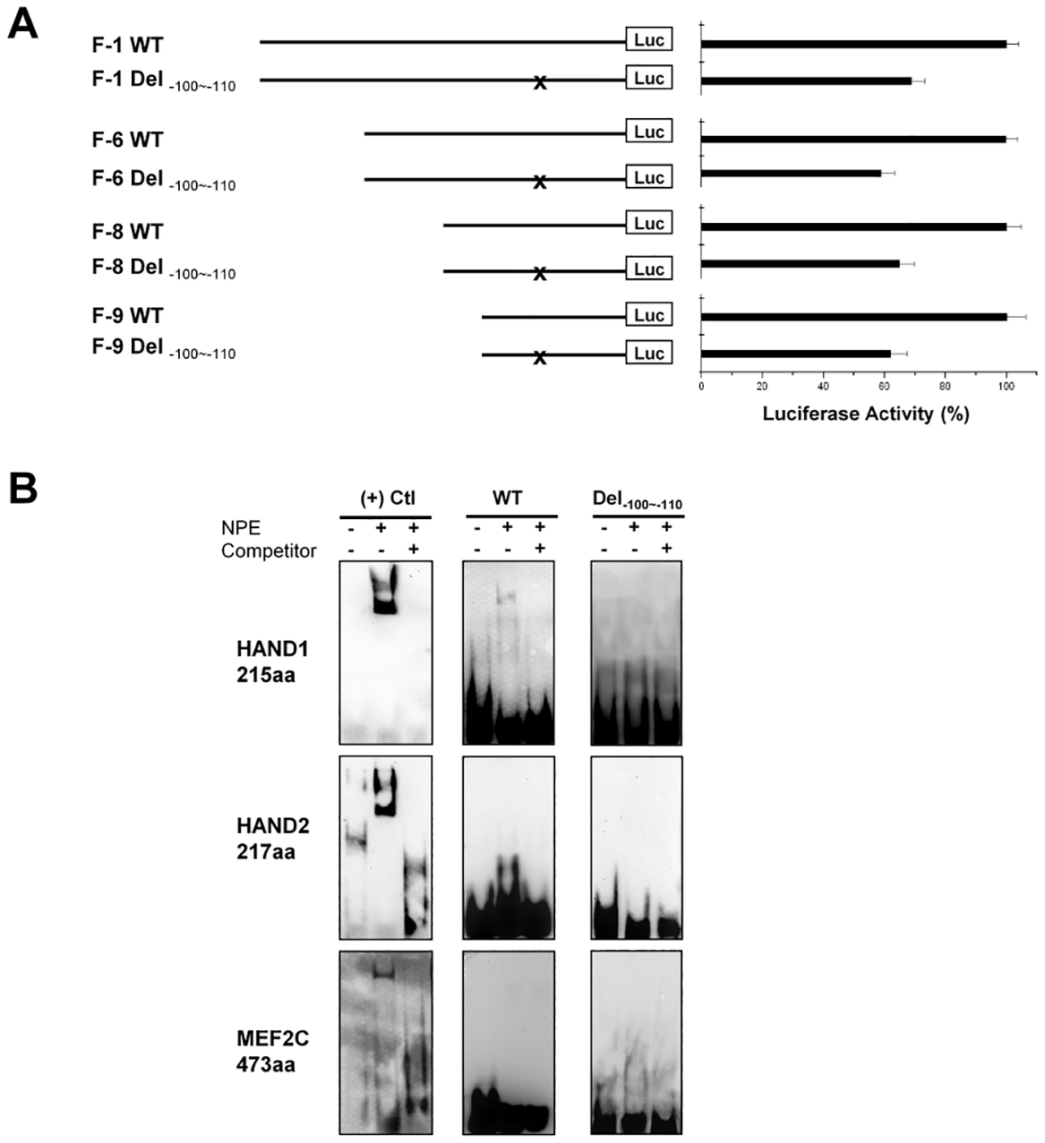
C._-100~-110_ caused a decrease in the promoter activity but the WT sequence of 11bp is neither an E-box nor a MEF2C binding site. Fig 4*A* shows that all F-1, -6, -8, and -9 containing homozygous c._-100~-110_ (*x*) demonstrated a decreased luciferase activity for about 30% as compared to each WT counterparts. Fig 4*B* illustrates EMSA results of HAND1 (*upper*), HAND2 (*middle*), and MEF2C (*lower panel*). Left rows show positive binding of HAND1, HAND2, and MEF2C to each of their positive control probes. Middle and left rows show negative bindings of three TFs respectively to WT probe and the probes containing c._-100~-110_. *n = 4*.

Next, we explored the impact of c._-100~-110_ on any potential binding of transcriptional factors to the region. Since c._-100~-110_ abolished the putative 3^rd^ E-box motif, we tested the impact on HAND1 and HAND2 binding. As shown in Fig 4, neither HAND1 nor HAND2 bound to the WT sequence or to the probe containing the c._-100~-110_. Furthermore, since c._-100~-110_ is an AT-rich region resemble a potential MEF2C binding motif, we tested MEF2C binding to this region. Unfortunately, we did not detect MEF2C binding to either WT sequence or the sequence containing the c._-100~-110_. Within the promoter region, all other loci of SNPs (A108T; C148G; A217G; G426C) did not markedly affect the promoter activity (S7 File).

### Normal Interaction of δ-SG-283R to DGC Components

**Fig 5:**
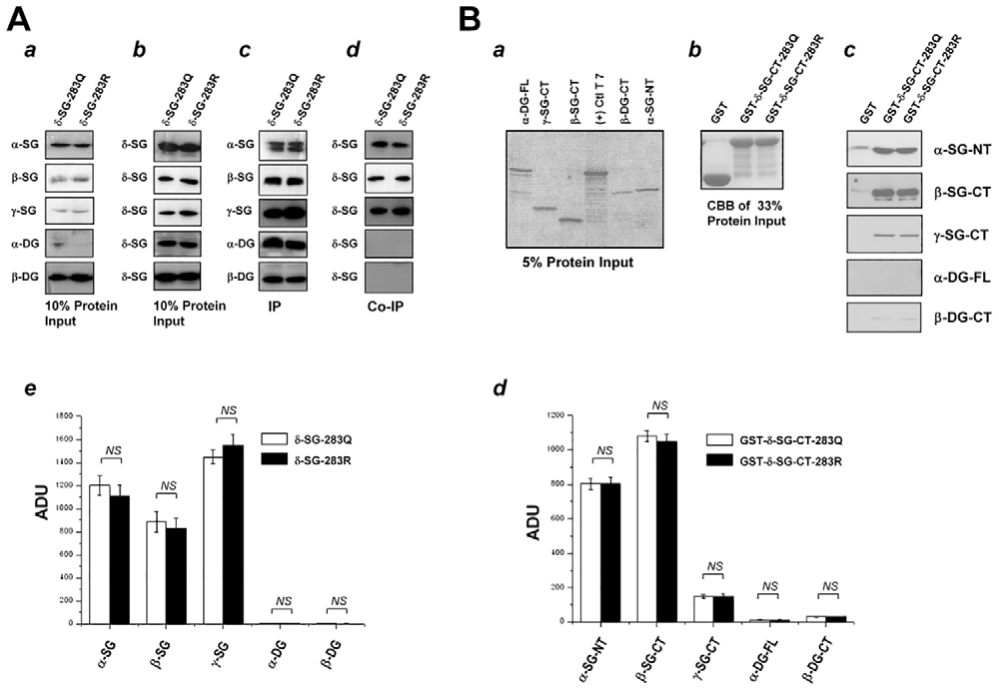
Comparison of δ-SG-283Q and δ-SG-283R on the interaction to other five DGC components. Fig 5*A* compares IP and Co-IP results between δ-SG-283Q and δ-SG-283R. In HEK293T cells, full-length δ-SG-283Q or δ-SG-283R was separately co-expressed with full-length α-SG, β-SG, γ-SG, α-DG, and β-DG. Then the cell were harvested and checked by WB for expression of each protein (*a*, *b*), IP (*c*), and Co-IP (*d*). A statistic summary of arbitrary unit of density (AUD) from 4 independent assays was shown (*d*). Fig 5*B* compares pull-down results between GST-δ-SG-CT-283Q and GST-δ-SG-CT-283R. 10% ^35^S-methionine-labeled protein input of α-SG-NT, β-SG-CT, γ-SG-CT, α-DG-FL, and β-DG-CT shown by autoradiography (*a*) and 33% protein input of GST, GST-δ-SG-CT-283Q, and GST-δ-SG-CT-283R shown by CBB staining (*b*) were used in the pull-down assay (*c*). The bound α-SG, β-SG, γ-SG, α-DG, and β-DG to GST-δ-SG-CT-283Q or to GST-δ-SG-CT-283R was compared by WB (*c*) with a statistic summary of AUD from 4 independent pull-down assays (*d*).

Since the WT residue 283Q shares homologous with β- and γ-SG (Fig 5C), and conversion of Q→R results in a dramatic change in electric charge, we thus tested its influence on its protein interactions with other five DCG components. We co-transfected 293T cells with full-length cDNA encoding each of the five DGC components in expression plasmids, and WT γ-SG or γ-SG-848A. Immunoprecipitation assays were performed using transfected whole cell lysate. The results showed that both WT δ-SG and δ-SG-848A strongly bind to β-SG and γ-SG with similar affinity, weakly to α-SG with similar affinity, and neither binds to α-DG nor β-DG (Fig 5A).

We further examined the effect of A848G on the direct association with the DGC components by pull-down assay. We expressed and purified both GST-δ-SG-CT-283Q (WT) and GST-δ-SG-CT-283R (SNP) from E. Coli (Fig 5A-a). The extracellular fragments of the indicated five components’ were obtained by *in vitro* translation (Fig 5A-b). *In vitro* protein pull-down assay demonstrated that both GST-δ-SG-CT-283Q and GST-δ-SG-CT-283R equally bind to α-, β-, γ-SG, but not to α- and β-DG (Fig 5A-c and 5A-d), similar to what we observed in immunoprecipitation assays.

### Association of δ-SG-283R with Mild Decrease in Plasma Membrane Localization of β/γ/δ-SG

**Fig 6:**
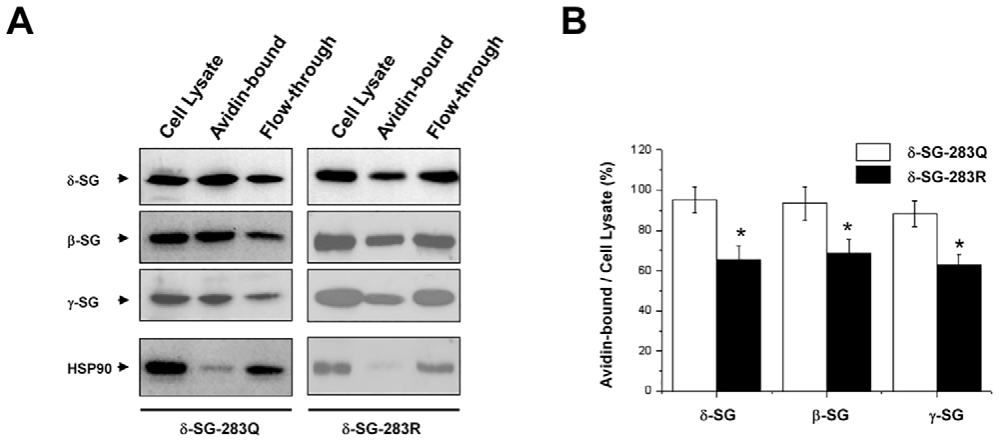
Effect of δ-SG-283R on Localization of β/δ/γ-SG to the Plasma Membrane Surface. Fig 6*A* compares cell surface biotinylation assay results between δ-SG-283Q and δ-SG-283R. In HEK293T cells, full-length δ-SG-283Q or δ-SG-283R was separately co-expressed with full-length β-SG and γ-SG. The amount of transfected sarcoglycans targeted to the cell surface was determined by labeling cells with membrane-impermeable NHS-LC-biotin. Biotinylated proteins were isolated with avidin and identified by WB. The specificity of cell surface biotinylation was demonstrated by the absence of cytoplasmic HSP70 in the avidin-bound fraction. In each lane, 10 μg protein was loaded. Fig 6*B* shows a statistic summary from 5 independent assays. *: *p*<0.05 *vs* δ-SG-283R.

It has been reported that β-SG and δ-SG were shown to form a core structure capable of targeting to the plasma membrane [29]. Since δ-SG-283R is located between 282C and 285N, whether it can change proper plasma membrane targeting? Above immunoprecipitation and pull-down results demonstrated that δ-SG interacts strongly with β- and γ-SG. Thus, we co-transfected wild-type β-SG and γ-SG with either WT δ-SG-283Q or δ-SG-283R in HEK-293T cells and performed cell surface biotinylation assay. We found that δ-SG-Q283R caused a mild but significant reduction of β/γ/δ-SG on the cell surface, as compared with WT δ-SG-283Q (Fig 6). These results indicate that δ-SG-283R has a mild detrimental effect on membrane localization of DGC.

## Discussion

DCM is a genetically heterogeneous disease and linkage analysis may be of limited value mainly because of the high mortality associated with the disease and the incomplete penetrance of disease mutations. So far almost all reported mutations for DCM have been found only in single families, and only few DCM-associated mutations have been detected in more than one patient [1, 3, 7–10]. Therefore, one of the key strategies to identify morbid genes is to screen for mutations in candidate genes in cohorts of sporadic DCM patients.

Based on previous reports, defects in the δ-SG gene seem to be unique and highly heterogeneous among DGC genes: 1) Six different LGMD-2F-causing mutations (E93X, A131P, R165X, E262K, c.656C, and W30X) demonstrated to be free of cardiac abnormalities [21, 30–33]. These mutations can be genetically transmitted in a mode of autosomal recessive. 2) Three different DCM-causing mutations (p.S151A, c.K238, and p.R71T) were not associated with the involvement of the skeletal muscle [18, 22]. Those mutations can be transmitted in a mode of autosomal dominant. 3) Deletion or mutation in the gene’s promoter can cause both DCM and HCM in both hamster and human being [17, 26, 34]. Those warrant our screening for genetic abnormalities in δ-SG gene in Chinese CM patients.

In the present study, we identified for the first time c._-100~-110_ and A848G polymorphisms in δ-SG gene. The co-occurred -/- of c._-100~-110_ and 848A/G is corrected with a significantly increased susceptibility of DCM. This is the fourth DCM-associated polymorphism/mutation reported in the δ-SG gene. Based on the fact that the responsible gene mutations reported so far could only account for about 30~35% of the DCM, our results further indicate that it is less likely that the remaining two-thirds mutations are all from new genes, but it is highly possible that a large part of new mutations will be from novel loci of those already-reported genes.

The genotype frequency of homozygous c._-100~-110_ was slightly higher in patients with CM. But due to a high prevalence of the allele in the normal controls, we postulated that it is a benign polymorphism that may have limited genetic significance. We tried two approaches to determine the functional relevance of the c._-100~-110_, luciferase reporter assay and EMSA. As shown by luciferase results, homozygous c._-100~-110_ unexpectedly caused a significant decrease in promoter activity. However, our EMSA results failed in demonstrating any positive binding of transcriptional factors to the region. So far, transcriptional regulations of the DGC complex genes remain unclear. HAND-1 and HAND-2 are important transcription factors (TFs) for cardiac muscle [35]. They share the same E-box motif (CANNTG) as MyoD, a key TF for skeletal muscle differentiation [36]. Among the DGC genes, α- and γ-SG are activated by MyoD [33, 37, 38]. The TFs that are responsible for controlling δ-SG gene transcription remain unknown, and possible candidates are HAND-1, HAND-2, MEF2C, Nkx2.5, NF-AT3, and CREB [39]. In our identified promoter region of the human δ-SG gene, we found two highly conserved putative binding motifs for MEF2C, one for Nkx2.5, and seven E-boxes. Based on our EMSA results, there was no evidence supporting that HAND-1 and HAND-2 are the TFs regulating human δ-SG gene transcription, nor the possibility that the sequence of c._-100~-110_ itself acting as a MEF2C binding site. Our mutagenesis results suggest that at least the 2nd E-box motif, which is located adjacent to c._-100~-110_, is one of the functional E-boxes. That c._-100~-110_ changes the downstream nucleotides of the 2nd E-box motif may contribute to the decreased promoter activity (Fig 3).

Despite a significant decrease in δ-SG promoter activity in the -/- of c._-100~-110_ polymorphism, there is no significant association of this polymorphism in patients with CM. On the contrary, the reported polymorphism -94G>C is associated with increased susceptibility of HCM in both the Japanese and Mexican population [25–27], the molecular mechanism remains to be unclear. It is possible that even with a mild to moderate decrease in δ-SG gene transcription, δ-SG protein translation could be compensated. Since c._-100~-110_ is not located in the 5’-UTR of the gene (Fig 4), it should not detrimentally affect post-transcriptional regulation of δ-SG mRNA stability and translation of the δ-SG protein [40]. The compensation need to be verified by measuring δ-SG gene transcription and translation in myocardial biopsy samples from subjects with -/- of c._-100~-110_.

On the other hand, the 848G allele seems to be sporadic in the Chinese and eastern Asia population. The genotype frequency of 848A/G was significantly higher in our patients with DCM (6.73%) as compared to HCM (1.38%) and normal controls (0.76%), suggesting the A848G is a rare polymorphism related to significantly increased susceptibility to DCM. Importantly, our result showed that when both homozygous c._-100~-110_ and heterozygous p.Q283R occurred coincidently in a single individual, the risk for DCM further increased to a surprisingly high level (OR = 39.85), indicating the pathogenic effects from both polymorphisms could be accumulative and amplified. Our results strongly suggest that the combination is one of the etiologies for human DCM in eastern Asia and the prevalence is 4.81% in our DCM patients, much higher than the previous reports where the mutation rate of δ-SG gene is less than 1.5% in both Europe and Japan [18, 21–24].

Furthermore, in this study all cases with the polymorphism are heterozygous. What cardiac phenotype will turn out to be in individuals who carry an 848G/G genotype? That may shed light on the role of the polymorphism in the pathogenesis of DCM. Because our patient pool is small, the prevalence of 848G/G is still unclear in DCM patient, but it could be much higher than it in the normal population. Based on our data, it is about 1.4/100,000 in the normal Chinese population. If individuals with 848G/G manifest a DCM phenotype, this number could account for about 7.4% of the etiology of the DCM, since the prevalence of DCM is 19/100,000 in China [41]. If added to the above 4.81% prevalence of the co-occurrence in DCM patients, these could account for >10% of the etiology of DCM, exceeding previously reported Lamin A/C gene mutations [7]. We are planning to expand our patient pool to sufficiently cover some cases of 848G/G to determine the cardiac phenotype.

Normal protein interactions among each DGC components are prerequisite for maintaining DGC normal architecture and function [29, 42, 43]. δ-SG is in close proximity to β- and γ-SG, especially their extracellular domains which consist of the same number of 231 amino acid (AA) residues [42]. The assembly of the complex follows a specific pathway: β- and δ-SG first form a core and then γ-SG acts as a linker to connect α-SG to the β/δ-SG core [29, 42, 43]. Those DGC proteins are divided into four functional domains, including intracellular, trans-membrane, extracellular proximal, and extracellular extreme C-terminus [43]. Their extracellular proximal domains are highly conserved and important for normal protein interactions among each DGC components [29]. Their extreme C-terminus domains constitute a trimeric β/δ/γ-SG complex with a symmetric EGF receptor-like structure, which might respond to an unidentified ligand or interact with a yet unidentified protein in the extracellular matrix [43]. Within the domains, there is a cysteine-rich motif that is supposed to form intramolecular disulfide bonds [29, 43]. The function of these disulfide bonds is still unclear and presumed to be important for their membrane localization. In case of δ-SG, two disulfide bonds are predicted to form between 264C and 289C and between 266C and 282C.

Mutations in the DGC genes can cause disruption of the complex and subsequently result in LGMD and/or DCM [12–16]. The mechanisms mainly involve in changes in protein localization, trafficking, protein-protein interaction, intramolecular disulfide bonds formation, and glycosylation. In cases of LGMD-2F-causing mutations reported so far, five (p.E93X, p.A131P, p.R165X, p.E262K, and c.656C) are located in the extracellular domain and only one (p.W30X) is located in the intracellular domain of the δ-SG protein [30–33]. In cases of DCM-causing mutations in the δ-SG gene, all three (p.S151A, c.K238, and p.R71T) are located at the extracellular domain [18, 21, 22]. p.S151A disrupted the nuclear localization of lamin A/C and emerin in cardiomyocytes due to nuclear sequestration of mutant δ-SG [44]. C.K238 is reported to disturb glycosylation of the protein. The p.R71T is located in the conserved region of the δ-SG gene and is supposed to alter the secondary structure of the protein by unknown mechanism [22].

The p.Q283R polymorphism is located between the disulfide bond-forming 282C and the glycosylation site of 285N. The WT residue Q is homologous to β- and γ-SG in the same location (Fig 4E). The conversion of Q→R results in a relatively dramatic change in the electric charge. As reported by Sakamoto *et al* [17], δ-SG binds to all other five DCG components with different affinity and we thus test the effect of 283R on its protein interactions with other five DCG components. Our results demonstrated that δ-SG binds to β-SG strongly, supporting that β-SG and δ-SG are fundamental to the assembly of the DGC. Our results also showed that δ-SG directly and strongly binds to γ-SG. However, the GST-δ-SG-CT-283R protein held a normal binding affinity with β- and γ-SG as GST-δ-SG-CT-283Q did, suggesting that p.Q283R is not affecting its interaction with β- and γ-SG. Instead, p.Q283R is associated with a mild reduction of β-, γ-, and δ-SG on the plasma membrane localization. Thus, it can cause disruption of the DGC and subsequently result in DCM [12–16]. *In vivo* effect of p.Q283R on the assembly of DGC needs to be evaluated in either DCM patients carrying 848G/G or in 848G/G-knock-in mouse model. In addition, p.Q283R may affect trimeric β/δ/γ-SG’s EGF receptor-like stereo-structure for some unidentified binding proteins in the extracellular matrix [29, 43]. Also, δ-SG is reported to be located in the terminal cistern of the sarcoplasmic reticulum independently of dystrophin and thus δ-SG-283R could be involved in the mechanism of the dysfunctional calcium signaling in DCM [45].

There are three limitations in this study. First, the size of the CM patient groups is small at a single center. The results might be affected due to bias of administered patients. Our findings need to be confirmed in a large cohort of DCM patients. Second, all of our patients samples were randomly collected and have no pedigree data. Therefore, the transmission mode of the co-occurred polymorphism is still unclear. Third, we lacked cardiac biopsy evidence in subjects with -/- of c._-100~-110_ to show the actual changes in δ-SG gene transcription and in the DCM patients with the co-occurred -/- of c._-100~-110_ and 848A/G to further show the mildly decreased plasma membrane localization of δ-SG-Q283R.

In conclusion, our results change our previous notion that the mutation frequency in the δ-SG gene is quite uncommon in patients with DCM, at least in eastern Asia. The co-occurred -/- of c._-100~-110_ and 848A/G could be used as a screening marker for genetic determination of DCM susceptibility in the regional countries.

## Supporting Information

S1 File Expanded Methods. (DOC)

S2 File S1 Table. Primers for Amplification of Genomic DNA Fragments for Human δ-SG Gene Promoter Assay.
(DOC)

S3 File S2 Table. Primers for Genomic DNA Amplification of 8 Exons of Human δ-SG Gene.
(DOC)

S4 File S3 Table. Probes for EMSA.
(DOC)

S5 File S4 Table. Haplotype Analysis in HCM Patients vs Control Population.
(DOC)

S6 File S1 Fig. Quality Control of Nuclear Protein Extract (NPE).
(DOC)

S7 File S2 Fig. Impacts of Other Single or Cluster of SNPs within the Promoter Region on the Promoter Activity.
(DOC)
