# Supplementary material for: A Haplotype of Two Novel Polymorphisms in δ-Sarcoglycan Gene Increases Risk of Dilated Cardiomyopathy in Mongoloid Population
Source: PLoS One. 2015 Dec 31;10(12):e0145602. doi: 10.1371/journal.pone.0145602 (PMC4697846; doi:10.1371/journal.pone.0145602)
Supplement: S1 File — (DOC) [file pone.0145602.s001.doc]

**Supporting Information file-1**

**Expanded Methods**

**Subjects and DNA Samples**

The study was approved by the institutional review board of Xinhua Hospital Affiliated to Shanghai Jiaotong University. Written informed consents for participation were obtained from all patients and healthy control subjects.

The patient pool consisted of 104 patients with DCM (65 men and 39 women, aged 17 to 68 years) and 145 with HCM (107 men and 38 women, aged 19 to 76 years). None of the patients had symptoms of limb-girdle muscular dystrophies. Diagnosis of CM was based on Contemporary Definitions and Classification of the Cardiomyopathies, established by American Heart Association ^1^. Patients with hypertension, coronary artery disease, valvular diseases, and congenital heart diseases were excluded. The control group included 790 unrelated healthy Chinese (427 men and 363 women, aged 21 to 65 years). The clinical and echocardiography data are summarized in Table I. Genomic DNA was extracted from whole peripheral blood with proteinase K digestion and the salting-out method. Genotyping data of 459 cases of normal Japanese DNA samples analyzed in the Institute of Medical Science of the University of Tokyo by Professor Kato were also included in this study. Some of the normal DNA samples, including 10 Japanese, 12 Koreans, 13 Thai, 110 Caucasian, and 110 Africans, were purchased from the Coriell Cell Repositories (Camden, NJ).

**Amplification of Genomic Fragments, Allele and Genotype Analysis, and Cloning**

As shown in Figure 1A, the genomic structure of the human δ-SG gene is established on the nucleotide sequence of human chromosome-5 clone CTB-56J15 (GenBank: AC027308.5: 270,154bp; NG_008693.2: 441,033bp) and human δ-SG cDNA (Genbank: variant-1: NM_000337; variant-2: NM_172244; variant-3: NM_001128209). The gene consists of 8 exons spanning more than 732kb (Chro-5: 155,462,147~156,194,799). Its 5’UTR consists of two regions, 5’UTR-1 is a 476bp area located 2301bp (interon-1) upstream of 5’UTR-2. 5’UTR-2, which is synonyms of the exon-1, consists of 46bp nucleotides and of them only the last 3bp ATG is the start codon for translation. There are two C-terminus spliced isoforms expressed in cardiac muscle as reported, which are only different in the exon-7 ^2, 3^.

Primers for amplification of the genomic fragments (F) and exons were designed from each intron-exon boundary (Supplementary Table 1 and 2) according to the structure. A high-fidelity polymerase KOD-FX-neo (Toyobo, Tokyo) was used in genomic DNA-templated PCR, which was performed in a 50ul volume, with initial denaturation at 94^0^C for 2 minutes followed by 30 cycles of 94^0^C for 30 seconds, 57^0^C for 45 seconds, and 68^0^C for 1~10 minutes (depending on F length). Each pair of primers yielded a single PCR band of the expected size. Obtained PCR products were then purified by phenol-chloroform and ethanol precipitation before subjected for sequencing or endonuclease (all from NEB, MA, USA) digestion and SYBR-Green (Molecular Probes) staining.

Sequencing results were analyzed by the software Sequencer 4.9 (Gene Codes Corp, Ann Arbor, MI, USA). Sequencing-identified normal F-1~10 and those fragments with newly-discovered polymorphism were cloned into the pRL3-basic firefly luciferase reporter vector between *Kpn* I and *Nhe* I site. In order to compare the E (E-box)-2, -3, and -6 and to avoid possible effects of two putative MEF2C binding motifs on the results, we utilized the shorter F-9 and mutated E-2 (CAGCTG🡪ACGCGT), -3 (CAGCTG🡪ACTGGC), and -6 (CACATGTG🡪AGACGTGT) by site-directed mutagenesis.

**Cell Culture**

A rat skeletal muscle cell line (L6, JCRB 9801), kindly gifted by Dr. Sakaki at the Institute of Medical Science of the University of Tokyo, and HEK-293T cells were cultured and passaged in Dulbecco’s modified Eagle’s medium (DMEM; Gibco-Life) with 10% fetal bovine serum (FBS; Gibco-Life), penicillin (100 units/ml), and streptomycin (100 ug/ml) at 37^o^C and 5% CO_2_.

**Transient Expression and Firefly Luciferase Reporter Assay**

L6 cells of 8~18 passages were seeded in 6-well plates at a density of 2×10^5^ cells per well 2 days prior to transfection. The cells were transfected in a serum-free DMEM with a mixture of 1μg of the pGL3-basic firefly luciferase reporter plasmid (Promega) with an insert of the F-1~12, 20ng of the pRL-SV40 Renilla luciferase internal control plasmid, and 3μl of FuGene (Roche Biochem) per well. pRL3-basic firefly luciferase reporter plasmid without insert and pRL3-basic firefly luciferase reporter plasmid with SV40 promoter were respectively transfected as negative and positive control. Twenty-four hours after transfection, the medium was changed to DMEM with 10% FBS. Cells were washed twice with phosphate-buffied saline (PBS) and harvested in 500μl of passive lysis buffer (Promega) 24 hours after the medium was changed. Then, luciferase activities were measured in three technical replicates with the dual-luciferase reporter assay system (Promega).

**Nuclear Protein Extraction (NPE)**

The left ventricular myocardial tissue was excised from a golden hamster, weighed, and rinsed in PBS. The tissue was pulverized using a tissue-tearor on ice, with supplemented 100x cocktail protease inhibitors (PI). The powder was immediately suspended in 10ml ice-cold PBS containing PI, mixed, and went through a cellular sifter (200-mesh size =76 μm; Baolan Standard Sieve Co., Shanghai). Flow-through was collected and the leftover large tissue debris were further homogenized and repeated the previous processes and collected. Flow-through was centrifuged at 800g for 15min and the supernatant was taken as the cytosolic fraction. The pellet was taken as individual or small clump of myocytes, which were further identified by immunocytostaining. The pellet was resuspended in 10 volumes of Buffer A (10mM HEPES, pH7.9; 10 mM KCl, 0.5 mM EDTA, 1mM DTT, 0.5 mM PMSF, 1xPI) and incubated on ice for 10 min, and mixed occasionally by flicking with a finger. The suspension was then centrifuged at 1850g for 15 min and the pellet was taken as an organelle and nuclear fraction. For every 100 μl of the pellet volume, 50 μl of Buffer B (20mM HEPES, pH7.9; 140mM NaCl, 10 mM KCl, 0.5 mM EDTA, 1% Triton-X-100, 1mM DTT, 0.5 mM PMSF, 1xPI) was added, mixed, and incubated on ice for 1 min. It was then centrifuged at 14,000g for 5 min, and the supernatant representing the organelle fraction was collected. The pellet was suspended with 5 volumes of Buffer C (25% glycerol; 20 mM HEPES, pH 7.9; 500 mM NaCl; 1.5 mM MgCl_2_; 0.5 mM EDTA, pH 8.0; 0.5mM PMSF, 1mM DTT, 1xPI), mixed, and incubated on ice for 40 min. It was then centrifuged at 14,000g for 20 min. The supernatant was taken as the NPE and the quality was checked by WB of Sp1 (Supplementary Table 3). The protein concentration was measured by Bradford method. The aliquot were snap frozen in liquid nitrogen and stored at −80°C until further analysis.

****Electrophoretic Mobilized Shift Assay (EMSA)****

Biotin-labeled or unlabeled DNA duplexes probes carrying MEF2C or E-box sequence (Supplementary Table 4) were made through annealing two reverse and complement single-stranded DNA. The NPE was pre-cleared with avidin-agarose (Thermo-Pierce 20219) to remove endogenous biotin. Binding reactions were performed by incubating 10 ug NPE for 20 min at room temperature with 2ug poly dI-dC (Amersham) and 200 fMol of each probes. DNA-protein complexes were resolved in 8% polyacrylamide (29:1 acrylamide: bisAcrylamide) gels and then transferred to Hybond-N^+^ membrane (Amersham, RPN303B). Unlabeled oligonucleotides in 200-fold excess were added as competitor control of the binding specificity. The further detection steps were performed using the Chemiluminescent Detection solution (Pierce, 34078).

**Cloning of Six DGC Components’ cDNA**

Each of six DGC components’ full-length cDNA was amplified from a human heart cDNA library (Clontech, CA) by a high-fidelity PCR. The PCR products were cloned into pcDNA3.1-myc/his(+)A between *EcoR*I-*Not*I (α-SG, β-SG, γ-SG, α-DG, and β-DG) or between *EcoRV*-*Sal*I (δ-SG) sites. For *in vitro* pull-down experiments, their intracellular and transmembrane domains were removed from each of their original constructs and the left length of the extracellular domains were shown as: α-SG-NT (870bp, AA 1-290), β-SG-CT (681bp, AA 93-318), γ-SG-CT (696bp, AA 61-291), and β-DG-NT (879bp, AA 1-293). The extracellular domain of -SG (702bp, AA 58-290) was sub-cloned into pGEX-4T-1 (Amersham Biosciences) between *Xho*I-*Not*I sites. The mutant cDNA of -SG-283R was obtained by site-directed mutagenesis of CGG from a wild type of CAG. Human Hand1, Hand2, and MEF2C’s cDNA were cloned in the same way into pQE-30 between *BamH*I and *Hind* III sites. All constructs were checked by sequencing.

****Antibodies****

The following mouse monoclonal antibodies were purchased from Novocastra (Newcastle upon Tyne, UK): α- (NCL-α-SARC); β- (NCL-β-SARC); γ- (NCL-γ-SARC); and -SG (NCL-δ-SARC); and β-DG (NCL-β-DG). Mouse monoclonal antibodies against α-DG (Clone VIA4-1) were purchased from Millipore (Upstate, NY, USA). Rabbit polyclonal antibodies against HSP90 were purchased from ABclonal (Wuhan, China).

**Immnuoprecipitation**

HEK-293T cells in 80–90% confluence were serum-starved for 12 h. After co-transfection of DGC components’ cDNA for 48 hours the cells were harvested in ice-cold TLB buffer, as described previously ^4-6^. The lysate was rocked at room temperature for 1h, and the supernatant was obtained by centrifugation. The supernatant underwent pre-absorption by protein-G beads for 1 h at 4 °C. After removal of the beads, the supernatant (500 μg of total protein) was mixed with target antibodies (3–4 μg) for 12 h rocking at 4 °C. Fresh protein-G beads were then added with a further 2-3h of rocking. Immunoprecipitates were centrifuged at 2500 rpm for 5 min at 4 °C, the supernatant was discarded, and the pellet was washed four times with TLB buffer. The pellet was then resuspended in 60 μl of SDS sample buffer.

***In vitro* Transcription-translation**

*In vitro* transcription and translation was performed using the TNT kit (Promega, WI) as directed by the manufacturer using 1ug of the expression construct plasmid (α-SG, β-SG, γ-SG, α-DG, and β-DG). ^35^S-methionine (Amersham Pharmacia Biotech; Cat. AG1049) was incorporated into nascent protein. For confirmation of the nascent protein expression, 5ul aliquot were removed and mixed with 15ul of SDS sample buffer. After heating at 100^o^C for 5 minutes, 10ul of the denatured sample was resolved by 10% SDS-PAGE and subjected to autoradiography (Figure 6A). Methionine with nonradioactive ^32^S-methionine labeling was adopted in the same reaction formula for translation and the protein samples were used for the following *in vitro* binding assay.

**GST- and 6His-fusion Protein Expression and Purification**

All GST- and 6His-fusion proteins were expressed in BL-21 E. Coli by induction of isopropyl-D-thiogalactopyranoside and purified through glutathione-sepharose beads (Sigma G4510) or Ni-beads (Qiagen, Cat-70666), as described previously ^4-6^.

**Pull-down Assay**

Fifty micro-liter reaction mixture containing 100ng of each translated α-, β-, and γ-SG and α- and β-DG proteins was mixed with 50 ul of GST-δ-SG-CT-283Q or GST-δ-SG-CT-283R bound to beads in 500ul TLB lysis buffer (20 mM Tris-HCl, 137 mM NaCl, 2 mM EDTA, 10% glycerol, 1% Triton X-100, 25 mM β-glycerol phosphate, pH 7.4) and rocked for 4h at 4°C. In control experiments, GST-alone beads were used. After a thorough washing with TLB lysis buffer, the bound proteins were solubilized in SDS sample buffer (125 mM Tris-HCl, 2% SDS, 20% glycerol, 10% 2-mercaptoethanol, pH 6.8) and boiled for 5 min, as described previously ^4-6^.

**Cell Surface Biotinylation (Our paper)**

Cultured HEK-293T cells were washed three times with PBS and treated with 1 mM membrane-impermeable NHS-LC-biotin (Thermo-Pierce 21335) in 50 mM HEPES (pH 7.4). After incubation at room temperature for 30 min, the reaction was quenched in 100 mM Tris-HCl (pH 7.4). The biotin-labeled cells were then washed three times with PBS and total proteins were extracted with TLB buffer, rocking at 4°C for 30min. The biotin-labeled proteins were isolated by incubating cell lysate (10 μg) with avidin-agarose (Thermo-Pierce 20219) for 2 h at 4°C. The flow-through fraction was collected by centrifugation at 10,000 × g for 1 min. The avidin beads were then washed with ice-cold PBS with 0.1% Tween-20 and the biotin-labeled proteins were eluted from the avidin beads with SDS sample buffer.

***Western* Blotting**

Samples were separated by SDS-PAGE (8~12% running, 4% stacking). The separated proteins were electrophoretically transferred to Immobilon-P with 0.45um pore size (Millipore, MA). Ponceau-S staining was utilized as a further assessment of loading and to check the fidelity of transfer. The blots were blocked for 1h at room temperature in PBS containing 0.05% Tween-20 (PBST) and 5% nonfat milk before probed with the primary antibodies (1:1000) overnight at 4 °C. After washing the membranes four times in PBST, a horseradish peroxidase-conjugated secondary antibody (1:2000; Amersham Biosciences) was added for 1 h. Immunodetection was accomplished using the ECL Western blotting detection kit (Thermo-Pierce) ^4-6^.

**Statistic Analysis**

Genotype frequencies in cases and controls were compared using a χ^2^- test with Yates' correction. Odds ratio (OR) and 95% confidence interval (CI) were calculated by unconditional logistic regression analysis. Linkage disequilibrium (LD) between c._-100~-110_ and A848G was estimated in the control group using Lewontin’s standardized coefficient *D’* and LD coefficient *r*^2^. The haplotypes were inferred from all genotype data, and haplotype analyses were conducted by using Haplo.stats (<http://www.mayo.edu/stagen>), which is a score test based on the generalized linear model framework by SNPStats (<http://bioinfo.iconcologia.net/snpstats/start.htm>) ^7^. All the statistical analyses were performed with the SPSS15.0 software, with two-sided tests and a significant level of *p* value <0.05.

**References**

1. Maron BJ, Towbin JA, Thiene G, Antzelevitch C, Corrado D, et al. (2006) Contemporary definitions and classification of the cardiomyopathies: an American Heart Association Scientific Statement from the Council on Clinical Cardiology, Heart Failure and Transplantation Committee; Quality of Care and Outcomes Research and Functional Genomics and Translational Biology Interdisciplinary Working Groups; and Council on Epidemiology and Prevention. Circulation 113: 1807-1816.

2. Tsubata S, Bowles KR, Vatta M, Zintz C, Titus J, et al. (2000) Mutations in the human delta-sarcoglycan gene in familial and sporadic dilated cardiomyopathy. J Clin Invest 106: 655-662.

3. Jung D, Duclos F, Apostol B, Straub V, Lee JC, et al. (1996) Characterization of delta-sarcoglycan, a novel component of the oligomeric sarcoglycan complex involved in limb-girdle muscular dystrophy. J Biol Chem 271: 32321-32329.

4. Wang YP, Ei-Zaru MR., Surks HK, Mendelsohn ME. (2004) Formin homology domain protein FHOD1 is a cyclic GMP-dependent protein kinase Ia binding protein and substrate in vascular smooth muscle cells. J Biol Chem 279: 24420-24426.

5. **Wang YP**, Zheng XR, Riddick N, Bryden M, Baur W,et al. (2009) **ROCK Isoform Regulation of Myosin Phosphatase and Contractility in Vascular Smooth Muscle Cells.** Circ Res 104: 531-540.

6. Chen J, McLean PA, Neel BG, Okunade G, Shull GE, et al. (2004) CD22 attenuates Ca2+ Signaling in B Cells by potentiating Plasma Membrane Ca2+ -ATPase activity. Nat Immunol 5: 651-657.

7. Solé X, Guinó E, Valls J, Iniesta R, Moreno V. (2006) SNPStats: a web tool for the analysis of association studies. Bioinformatics 22: 1928-1929.
