# Supplementary material for: A Haplotype of Two Novel Polymorphisms in δ-Sarcoglycan Gene Increases Risk of Dilated Cardiomyopathy in Mongoloid Population
Source: PLoS One. 2015 Dec 31;10(12):e0145602. doi: 10.1371/journal.pone.0145602 (PMC4697846; doi:10.1371/journal.pone.0145602)
Supplement: S3 File — Primers for Genomic DNA Amplification of 8 Exons of Human δ-SG Gene. (DOC) [file pone.0145602.s003.doc]

**Supporting Information file-3**
