# Supplementary material for: A Haplotype of Two Novel Polymorphisms in δ-Sarcoglycan Gene Increases Risk of Dilated Cardiomyopathy in Mongoloid Population
Source: PLoS One. 2015 Dec 31;10(12):e0145602. doi: 10.1371/journal.pone.0145602 (PMC4697846; doi:10.1371/journal.pone.0145602)
Supplement: S6 File — Quality Control of Nuclear Protein Extract (NPE). (DOC) [file pone.0145602.s006.doc]

**Supporting Information file-6**

**S1 Fig: Quality Control of Nuclear Protein Extract (NPE).** Figure illustrates an EMSA result of Sp1 binding to its positive control probe.
