# Supplementary material for: A Haplotype of Two Novel Polymorphisms in δ-Sarcoglycan Gene Increases Risk of Dilated Cardiomyopathy in Mongoloid Population
Source: PLoS One. 2015 Dec 31;10(12):e0145602. doi: 10.1371/journal.pone.0145602 (PMC4697846; doi:10.1371/journal.pone.0145602)
Supplement: S7 File — Impacts of Other Single or Cluster of SNPs within the Promoter Region on the Promoter Activity. (DOC) [file pone.0145602.s007.doc]

**Supporting Information file-7**

**S2 Fig: Impacts of Other Single or Cluster of SNPs within the Promoter Region on the Promoter Activity.** Figure shows WT F-9 and those F-9 with other single (G426C) or cluster of SNPs (A108T; C148G; A217G; G426C) and their corresponding luciferase activities.
